# Supervisory styles and graduate student innovation performance: The mediating role of psychological capital and the moderating role of harmonious academic passion

**DOI:** 10.3389/fpsyg.2022.1034216

**Published:** 2022-11-03

**Authors:** Bingbing Yang, Shuimei Bao, Juan Xu

**Affiliations:** ^1^School of Education, Huazhong University of Science and Technology, Wuhan, China; ^2^Institute of Higher Education, Lanzhou University, Lanzhou, China; ^3^School of Education Science, Ludong University, Yantai, China

**Keywords:** supervisory styles, graduate student, innovation performance, psychological capital, harmonious academic passion

## Abstract

Supervisory styles Are Key predictors of graduate students’ innovation performance (GSIP), but the mediating and moderating mechanisms underlying this relationship require further exploration. Based on the job demands-resources model and conservation of resources theory, this study analyzed the influence of supervisory styles on GSIP, including the mediating role of psychological capital (PsyCap) and the moderating role of harmonious academic passion (HAP). Questionnaires were completed by 400 graduate students from a Chinese university. The results indicated that (1) both supportive and directive supervisory styles (SSS and DSS) were positively related To GSIP, (2) PsyCap fully mediated the relationship between SSS and GSIP, and (3) HAP significantly moderated the effect of DSS but exhibited no moderating influence on the effect of SSS. These findings contribute to a deeper understanding of why, how and when supervisory styles influence GSIP. Implications for both theory and practice as well as the limitations of this research are discussed.

## Introduction

Over the past decade, China has implemented a number of measures to improve the quality of postgraduate education and to develop innovative capacity (Liu et al., 2020). However, several large-scale surveys on the quality of postgraduate training in China since 2000 have consistently revealed that the overall situation of postgraduate innovation in China is not encouraging. Furthermore, on the one hand, due to the popularization of higher education in China, the number of graduate students is increasing rapidly. On the other hand, the delayed graduation rate of doctoral students in China is increasing, and a lack of academic output has emerged as the most significant barrier to graduation. Therefore, improving graduate students’ innovation capability and performance has become an important subject in Chinese graduate education research.

In China, the supervisor responsibility system is the main approach used to graduate education, and the supervisor is the person most responsible for training postgraduates (Wang et al., 2022a). Al-Sawai (2013) defined leadership as “the behavior of an individual directing the activities of a group toward a shared goal.” Thus, the behavior associated with and process of supervising postgraduate students also constitute a form of leadership. Postgraduate education in China is currently plagued by issues such as a “*laissez-faire*” approach, “squeezing guidance,” and insufficient guidance, thus indicating the urgency of research concerning supervisor leadership (Bao and Yang, 2021). Numerous organizational studies have explored the relationship between leadership and employee creative and innovative performance (Hughes et al., 2018; Lee et al., 2019; Syed et al., 2021). However, leadership research in the education sector has typically focused on executive positions (Al-Husseini and Elbeltagi, 2016; Akhtar et al., 2021; Li et al., 2022) and has given less attention to academic leadership (Zacher and Johnson, 2015; Meng and Zhao, 2018) and its effect on students’ creativity (Gu et al., 2017; Meng et al., 2017).

The topics of supervisor leadership and graduate students’ creativity and innovation have drawn a great deal of attention in recent years (Liu et al., 2020). Researchers have claimed that supervisors’ leadership has both direct and indirect impacts on graduate students’ innovation and performance (Gu et al., 2017). For instance, intrinsic motivation mediates the relationship between supervisors’ leadership and graduate students’ creativity (Zacher and Johnson, 2015; Gu et al., 2017; Meng et al., 2017; Meng and Zhao, 2018; Xia et al., 2021). Additionally, creative self-efficacy (Gu et al., 2017) and professional knowledge (Meng and Zhao, 2018) have been shown to mediate the relationship between leadership style and innovation. However, it is obvious that previous research has overemphasized the mediating effect of intrinsic motivation to the detriment of other variables (Hughes et al., 2018). Researchers have also begun to outline the boundary conditions of the effect of supervisors’ leadership on graduate students’ innovation. For instance, personal initiative may serve as a moderator in the relationship between supervisors’ leadership and graduate students’ innovation (Wu et al., 2018). While previous research has provided useful insights into the mediating mechanisms and boundary conditions associated with the effect of supervisor leadership on graduate students’ innovation, additional research is required to uncover the dynamics through which supervisor leadership influences graduate students’ outcomes (Xia et al., 2021).

NATURE PhD SURVEY 2019 reported that 36% of respondents have sought help for anxiety or depression (Je, 2019), indicating that attention should be given to the psychological health of graduate students. Psychological capital (PsyCap), which is defined as an individual’s positive psychological state of development, can be developed and managed to promote performance enhancement (Luthans et al., 2007a). Numerous empirical studies have confirmed the strong relationship between PsyCap and employee attitudes, behaviors, and performance (Newman et al., 2014). Further, the mediating role played by PsyCap especially in the relationship between organizational environments and employee outcomes has been examined (Newman et al., 2014). Despite the fact that PsyCap has also been studied in educational contexts (Guo et al., 2021), little is known regarding graduate students’ PsyCap and its role as a mediator in the relationship between supervisor leadership and graduate student innovation performance (GSIP). The development of innovation among graduate students does not occur in a psychological vacuum (Liu et al., 2020). Accordingly, this study employs PsyCap as a mediating variable in the relationship between supervisor leadership and GSIP in response to calls for further exploration of mediating variables other than intrinsic motivation (Hughes et al., 2018).

Previous research on the boundary conditions associated with the effect of supervisor leadership on graduate students’ innovation has been limited, and the potential moderating effects of numerous individual characteristics have not been considered (Zacher and Johnson, 2015; Meng et al., 2017). In situational leadership theories, individual characteristics may impact the effects of leadership (Hersey and Blanchard, 1982). Harmonious academic passion (HAP) is a crucial personal trait that motivates graduate students to conduct research willingly, and studies have shown that passion has a significant impact on performance (Vallerand et al., 2007). Answering the recent calls mentioned above, this study further examines the ways in which supervisory leadership and HAP interact to affect GSIP. Research on supervisor leadership and graduate students’ creativity has employed theoretical perspectives drawn from social cognitive theory (Gu et al., 2017) and Amabile’s componential theory of creativity (Meng and Zhao, 2018) to explain the mechanism underlying the relationship between these two factors, neglecting other theoretical explanations. The study employs the job demands-resources (JD-R) model and conservation of resources (COR) theory as distinct theoretical perspectives to clarify why, how and when supervisor leadership affects GSIP.

**Figure 1:**
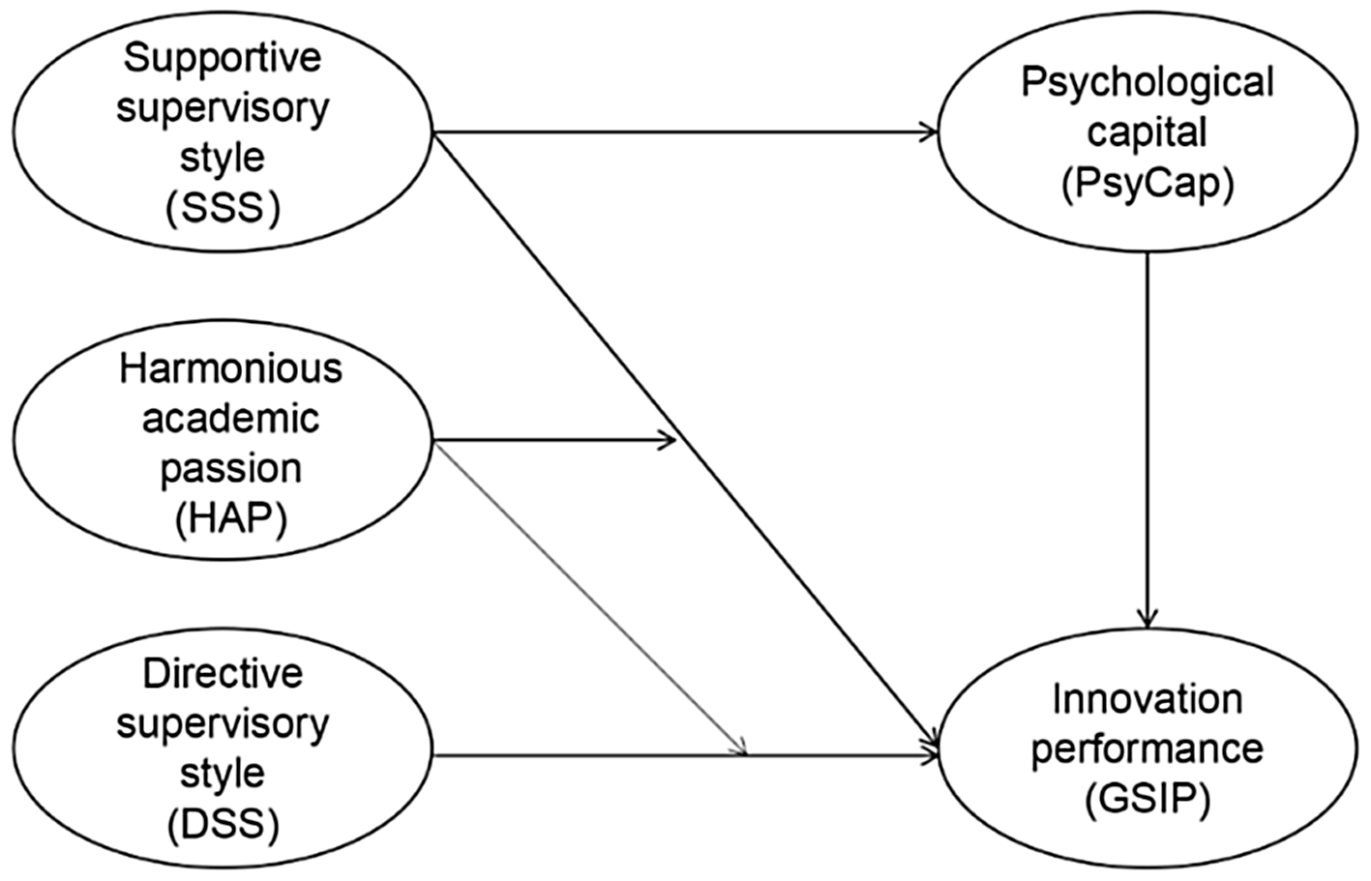
Research model.

The current study adds to the literature on leadership and innovation performance by investigating academic leadership and its impact on student outcomes in the education sector. First, by integrating PsyCap as a psychological mechanism, the current study expands the literature on the relationship between supervisor leadership and GSIP. Second, by examining HAP’s moderating effect, the study contributes to the understanding of boundary conditions for supervisor leadership on GSIP. Finally, by introducing the JD-R model and COR theory, the theoretical framework for the influence of supervisor leadership on the academic development of graduate students is expanded. The research model is shown in Figure 1.

## Theory and hypothesis development

The JD-R model and COR theory constitute way-of thinking about the impacts of job demands and job resources (Demerouti et al., 2001; Schaufeli and Bakker, 2004) as well as personal characteristics (Xanthopoulou et al., 2007) on employee psychological states and outcomes. Job demands might lead to resource loss, which can result in stress, health problems or other negative outcomes. Job resources are especially important for resource gain, which is in turn important for well-being or other positive outcomes. Following a method similar to that described above, the current study investigates the effect of job characteristics (supervisor leadership) and personal resources (PsyCap, HAP) on the outcome variable (GSIP) in an academic context.

### Supervisory styles and innovation performance

According to the literature (Wang, 2013; Bao and Yang, 2019), supportive and directive supervision constitute the two fundamental supervisory styles used in China. This finding is consistent with the conclusions of previous studies on leadership, which have differentiated leadership into supportive and directive supervisory styles (Gu et al., 2017). A supportive supervisory style (SSS) occurs when a supervisor behaves in ways that favor relationship building with an emphasis on meeting the needs and preferences of students, caring for their well-being, and fostering a friendly and comforting research atmosphere (Gu et al., 2017). Normally, this supervisory relationship includes a combination of three types of support: (1) personal support, such as providing emotional support and boosting confidence when students face obstacles; (2) academic support, including being available to help with academic activities and providing timely feedback on student progress; and (3) autonomy support, e.g., recognizing the student’s viewpoint, urging them to express their thoughts openly, and giving them the opportunity to make their own decisions (Overall et al., 2011). A directive supervisory style (DSS), in contrast, primarily reflects task-oriented behavior by a supervisor that aims to provide team members with a framework for decision-making and action that is in line with the supervisor’s vision (Somech, 2016).

In the present study, innovation performance can be divided into “innovation” and “performance.” Innovation is commonly defined as the production or adoption of useful ideas and idea implementation (Scott and Bruce, 1994). As a core attribute of graduate students, innovation is particularly important to reach innovative research achievements. Performance represents the output that is made visible and known to others (Zhao et al., 2021b). When examining the process holistically, innovation performance is defined as a construct comprising an innovation process that is similar to innovative research behavior (Janssen, 2000) and an innovation outcome that is similar to academic research output (Guo et al., 2021). Previous research has examined a variety of individual and contextual factors as potential predictors of innovative work behavior and performance (Afsar et al., 2014; Etikariena, 2016). Among these factors, leadership and positive psychological states have proven to be the most influential (Kim and Beehr, 2022). Positive leadership behaviors such as supportive leadership, empowering leadership, and inclusive leadership, are positively related to employees’ innovative work behaviors and task-related performance (Gupta and Singh, 2014; Fang et al., 2019; Wang et al., 2022b).

Based on COR theory, supportive leadership can be viewed as a critical resource for employees in the workplace (Demerouti et al., 2001), which is effective in achieving positive results. Accordingly, the present study proposes that SSS could enhance GSIP. First, by providing personal support, supervisors offer graduate students resources to achieve their goals by providing them with reassurance and empathy, which can support them when they are faced with research-based obstacles, personal stressors, and confidence crises (Overall et al., 2011). This display of confidence plays an important role in reinforcing positive self-image and fostering positive work outcomes. Second, students who receive academic support from their supervisors obtain direct task-related assistance, such as help with research-related skills and practical issues. This type of support is a critical resource that allows students to advance in the research process (Amabile et al., 2004; Gupta and Singh, 2014). Finally, autonomy support constitutes a job resource that satisfies students’ need for autonomy (Ryan and Deci, 2000); it can improve graduate students’ enthusiasm, increase their autonomous motivation (Meng and Zhao, 2018), thus contributing to innovative performance. Overall, in line with COR theory and the JD-R model, SSS provides empathy, autonomy, feedback, advice, and practical assistance, all of which can aid students in engaging in innovative behaviors and yield better performance. Accordingly, the following hypothesis is presented:

*H1a*: SSS is positively related to GSIP.

DSS involves behavior by a supervisor that is focused on guiding task completion, managing debates, and dominating interactions (Gu et al., 2017). According to the JD-R model, DSS tends to be considered a challenge (Crawford et al., 2010) in Chinese higher education due to the culture of China, which emphasizes collectivism and high power distance (Gu et al., 2017). Subordinates who are accepting of this type of hierarchical power structure are more inclined to believe that leaders have inherent superiority, authority, and status (Peltokorpi, 2018). Because China is a traditional society that features high power distance (Hofstede, 1984), in the context of Chinese higher education, graduate students tend to accept DSS. Indeed, Chinese graduate students are more likely to take DSS for granted and evaluate this type of relationship with their supervisors as less stressful. Furthermore, DSSs that involve strict deadlines, for example, can shift graduate students’ attention away from nonlearning processes and toward problem solving. That is, DSS reduces cognitive “bad load” (Gu et al., 2017). Previous studies have demonstrated that directive leaders help their followers resolve tasks, clarify ambiguous roles, provide external monitoring, and reduce process loss, eventually leading their subordinates to perform at a higher level (Lorinkova et al., 2013; Somech, 2016). Thus, the following hypothesis is proposed:

*H1b*: DSS is positively related to GSIP.

### Mediation of PsyCap

PsyCap, which is defined as an individual’s positive psychological state of development as manifested through self-efficacy, hope, optimism and resilience (Luthans et al., 2007a), can be developed and managed to promote performance enhancement (Luthans and Youssef, 2004). Self-efficacy is defined as “believing in one’s ability to mobilize cognitive resources to obtain specific outcomes”; hope refers to “having the willpower and pathways to attain one’s goals”; optimism refers to “the explanatory style that attributes positive events to internal, permanent and pervasive causes”; and resilience is “the capacity to bounce back from adversity, failure or even seeming overwhelming positive changes” (Luthans and Youssef, 2004). Although each of these four positive psychology constructions can improve employee outcomes, the higher-order factor may be better predictors of outcomes than the four individual facets (Luthans et al., 2007a). The organizational environment and, in particular, leadership are the primary antecedents of PsyCap. Previous research has identified various types of positive leadership that are conducive to the development of employee PsyCap, such as inclusive leadership, ethical leadership, transformational leadership, and authentic leadership (Rego et al., 2012; Bouckenooghe et al., 2014; Fang et al., 2019; Lei et al., 2020). There is a broad consensus among researchers that supportive leadership behaviors can boost employees’ PsyCap. However, research on supervisor leadership and graduate students’ PsyCap remains scarce (Ahmed et al., 2017).

According to COR theory, people strive to maintain and accumulate resources of various kinds, including job resources such as supervisory support. Therefore, SSS can improve the pool of resources from which students can draw, and this resource gain can help students develop a positive psychological state. First, by offering academic support, such as by providing task-related help, supervisors can help students obtain the knowledge and skills that they need to conduct scientific research more quickly and directly, thus making them more competent. Guo et al. (2021) discovered that the more competitive a student is, the greater their PsyCap. Second, supervisors, by providing autonomy, support and developmental feedback create an environment of self-determination, security and trust that enables students to concentrate their efforts on goal-related tasks and on the task of finding alternative pathways to solve problems and benefit from opportunities (Rego et al., 2012). Finally, when supervisors provide personal support to students by comforting them and empathizing with them, students are able to quickly recuperate from setbacks, which increases their resilience. By expressing respect and confidence in the student’s competence and talents, supervisors can help students see the positive side of situations and shift their emphasis away from the negative aspects, which can increase students’ resilience (Luthans et al., 2007b). As a job resource, SSS fosters the emergence of PsyCap in graduate students.

Several empirical studies have found evidence to support such an inference. Overall et al. (2011) discovered that autonomy support was an indicator of greater research self-efficacy. Gu et al. (2017) found that SSS was positively related to graduate students’ creative self-efficacy. Although limited, Ahmed et al. (2017) demonstrated that supervisor support is positively related to postgraduate students’ PsyCap. Thus, the following hypothesis is proposed:

*H2*: SSS is positively related to graduate students’ PsyCap.

PsyCap is often defined as a set of personal resources (Ahmed et al., 2017) that can help people achieve work objectives and personal growth in the same manner as job resources (Schaufeli and Taris, 2014). Numerous studies have examined the impact of PsyCap and its various facets on employee attitudes, behaviors and performance (Newman et al., 2014). Building on existing research as well as by reference to COR theory, the present study proposes that graduate students’ PsyCap is positively related to their innovation performance. It is well known that the research process is not always smooth; it may be associated with risks and uncertainties, and it consumes students’ valuable resources (e.g., time, energy, self-confidence, and optimism), thus causing them to feel stressed and tense. PsyCap offers students a positive psychological resource that allows them to cope with stress (Li et al., 2015). Graduate students with high levels of PsyCap: (1) believe in their abilities to mobilize the motivation, cognitive resources, and courses of action necessary to conduct academic research successfully (self-efficacy), have the willpower and pathways necessary to achieve their research objectives (hope), make positive attributions of research difficulties and failures (optimism), and can bounce back from experimental failures or even seemingly overwhelming positive changes (resilience) (Luthans and Youssef, 2004). In summary, students with high levels of PsyCap have more resources at their disposal that allow them to engage in academic innovation and exhibit improved performance. Guo et al. (2021) found that postgraduates’ PsyCap is positively associated with their academic research performance. Thus, the following hypothesis is proposed:

*H3*: PsyCap is positively related to GSIP.

According to the relationships discussed above, it is possible that PsyCap mediates the relationship between SSS and GSIP. That is, supervisor support can improve graduate student PsyCap and thus lead to high innovation performance. According to COR theory, employees who work in a resourceful environment tend to develop personal resources that facilitate positive outcomes. The JD-R model also implies that personal resources mediate the relation between job characteristics and well-being (Schaufeli and Bakker, 2004). Empirically, studies have found that PsyCap mediates some types of the effect of leadership on employees’ work outcomes (Rego et al., 2012; Bouckenooghe et al., 2014; Gupta and Singh, 2014). Limited evidence has also suggested that research performance and postgraduate competence are partially mediated by PsyCap (Guo et al., 2021). Thus, the following hypothesis is proposed:

*H4*: PsyCap mediates the positive relationship between SSS and GSIP.

### Moderation of HAP

Academic passion can be understood as an individual’s strong inclination toward academic research that the individual loves, values highly, and engages in regularly (Vallerand, 2015). Specifically, HAP results from autonomous internalization, which refers to graduate students’ free acceptance that academic research as important for them without any contingencies (Liu et al., 2016). HAP is a motivational force that leads graduate students to engage willingly in academic research (Bélanger and Ratelle, 2021). Previous research has demonstrated that HAP is positively related to academic engagement (Zhao et al., 2021a) and academic thriving (Zhou, 2021).

According to the JD-R model, HAP is a personal resource that can exacerbate the positive effect of SSS on GSIP (Schaufeli and Taris, 2014). SSS is aligned with HAP, and this match contributes to graduate students’ optimal functioning (van den Broeck et al., 2011). This is because students with high HAP attempt to use available resources (such as supervisory support), which could assist them in achieving their goals. This assumption is also consistent with the contention that a match between personal and job characteristics can result in positive outcomes (Parkes, 1994). Thus, the following hypothesis is proposed:

*H5a*: HAP strengthens the positive association between SSS and GSIP; that is, this relationship is stronger when the level of HAP is high.

H1b predicts that DSS is positively related to GSIP. However, DSS may be incompatible with the leader behavior expected by harmoniously passionate students. That is, for students with high HAP, the positive effect of DSS on GSIP is lessened. Specifically, students with high HAP exhibit more academic initiative, and they are accustomed to self-directed goals rather than assigned goals. Thus, they may be uncomfortable with or may even reject DSS. Conversely, students who lack HAP have no clear research plan and are more likely to accept tasks assigned by their supervisors. It has been demonstrated that students’ initiative negatively moderates the positive relationship between controlling instructions given by a supervisor and students’ innovative thinking and behavior (Wu et al., 2018). Thus, the following hypothesis is proposed:

*H5b*: HAP lessens the positive association between DSS and GSIP; that is, this relationship is stronger when the level of HAP is low.

## Materials and methods

### Sample and procedures

The present study employed a cross-sectional research design and used the convenience sampling method to collect data. In this study, participants consisted of graduate students from a university in Lanzhou, China. This “double first-class” university was permitted to establish a graduate school in 2004, and graduate students now account for more than 45% of the total enrolment. Hence, this university constituted an excellent location for the survey. First-year master’s students were excluded from the study because they had yet to demonstrate clear innovative performance. A senior administration officer from the graduate school was contacted to assist with the survey. He assisted in forwarding the questionnaire’s hyperlink to the administration in each college, which then notified the students to respond. Data were collected between 5 November 2019 and 23 November 2019.

In this study, 459 questionnaires were gathered, 400 of which were valid; therefore, the effective rate of return for the questionnaire was 87.15%. Among the final participants, 33% were men, 67% were women, 78.5% were master’s students, 21.5% were doctoral students, 64.5% studied in the sciences and technology, and 35.5% were students in the humanities and social sciences.

### Measures

All scales used in the research are mature. SSS, DSS, HAP, and GSIP scale items are scored on a 5-point Likert scale ranging from 1 (strongly disagree) to 5 (strongly agree). PsyCap scale items are rated on a 7-point Likert scale ranging from 1 (strongly disagree) to 7 (strongly agree). The scales used in this study all have good reliability and validity (Table 1).

**Table 1:** Reliability and validity of the scales used in this study.

Variables	Validity						Reliability
	*χ*^2^/*df*	GFI	RMSEA	CFI	TLI	SRMR	Cronbach’s *α*
SSS	3.14	0.95	0.07	0.98	0.98	0.04	0.96
DSS	1.59	0.99	0.04	0.99	0.99	0.02	0.89
HAP							0.92
PsyCap	2.87	0.94	0.07	0.97	0.96	0.03	0.94
GSIP	2.28	0.98	0.06	0.99	0.99	0.02	0.90

Since both the DSS and the HAP scales were unidimensional and the HAP scale has only 3 items, they were used as a two-factor model in this study for CFA.

#### SSS

A 10-item scale adapted from Overall et al. (2011) was used to measure SSS, which includes three dimensions: autonomy support (3 items), academic support (3 items), and personal support (4 items). This scale has been used to investigate Chinese graduate students in prior research (Gu et al., 2017). A sample item is “My supervisor encourages me to ask questions.”

#### DSS

DSS was measured using a four-item scale borrowed from Wang (2013). A sample item is “My supervisor sets the goals for my research performance.”

#### PsyCap

Thirteen items adapted from the PsyCap questionnaire (PCQ) were used to measure graduate students’ PsyCap. Self-efficacy (3 items), hope (3 items), resilience (4 items), and optimism (3 items) were the four components of the scale. A sample item is “When faced with uncertainty in my studies, I usually hope for the best.”

#### HAP

HAP was measured using the graduate student academic loyalty questionnaire subscale (Cao et al., 2008). It contained three items, such as “I am interested in academic research.”

#### GSIP

GSIP was operationalized as encompassing two constructs: the innovation process (3 items) and innovation outcomes (4 items). Items for each construct were borrowed from validated and reliable instruments used in previous research (Scott and Bruce, 1994; Chen and Li, 2018). A sample item is “Develops adequate plans and schedules for the implementation of new ideas.”

### Data analysis

First, because the data were self-reported, several procedural remedies were used to determine whether the results were seriously threatened by common method bias (CMB). Second, descriptive statistics and Pearson correlations were performed on the preliminary analyses using SPSS 26. Third, AMOS 21 was used to perform confirmatory factor analysis (CFA) to test the measurement model and provide maximum likelihood estimates for the four suggested components. Fourth, AMOS 21 was used to conduct structural equation modelling (SEM) to assess the mediating effect of PsyCap on the relationship between SSS and GSIP. Specifically, the indirect effect was estimated using a bootstrapping approach with 5,000 resamples (Preacher and Hayes, 2008). Finally, PROCESS Model 1 (Hayes, 2017) was used to examine the moderating effect of HAP on the relationship between supervisory styles and GSIP. Additionally, a simple slope analysis was conducted.

## Results

### Preliminary analyses

Because the data were self-reported, several procedural remedies were used to minimize common method bias (CMB). First, participation was entirely voluntary, and respondents were guaranteed confidentiality and anonymity. Second, Harman’s single-factor test revealed that the first factor in the exploratory factor analysis accounted for 39.36% of the variance, which was less than the critical standard of 40.0% (Podsakoff et al., 2003). Third, CFA was conducted (Malhotra et al., 2006). An internal consistency approach (Kishton and Widaman, 2016) was used to create parcels for SSS, PsyCap and GSIP. For example, three parcels were constructed for SSS using its different facets as grouping criteria: autonomy support, academic support, and personal support. These parcels were treated as indicators of their respective latent variables in the CFA. The CFA results confirmed that the one-factor model exhibited a poorer data fit (*χ*^2^ = 2340.58, RMSEA = 0.23, SRMR = 0.18, CFI = 0.53, GFI = 0.45) than the five-factor model (*χ*^2^ = 185.42, RMSEA = 0.05, SRMR = 0.03, CFI = 0.98, GFI = 0.94). Therefore, CMB should not be a concern in this research. Finally, researchers have indicated that the likelihood of CMB is lower in studies featuring a moderator because respondents find it difficult to predict the moderating effect (Simons and Peterson, 2000).

As indicated in Table 2, SSS, DSS, HAP, PsyCap and GSIP were all positively associated (r ranged from 0.28 to 0.72). All the correlations among the variables were significant at the 0.01 level, providing preliminary evidence for further hypothesis testing.

**Table 2:** Means, standard deviations, and correlations (*n* = 400).

Variables	Means	*SD*	1	2	3	4
1. SSS	4.23	0.79				
2. DSS	3.89	0.88	0.72^**^			
3. HAP	3.58	0.87	0.34^**^	0.30^**^		
4. PsyCap	5.15	0.94	0.36^**^	0.28^**^	0.65^**^	
5. GSIP	3.30	0.70	0.32^**^	0.32^**^	0.55^**^	0.66^**^

** *p* < 0.01 (two tailed).

### Measurement model

As shown in Table 3, the proposed four-factor structure performed significantly better than the five alternative models in terms of data fit. The fit indices supported the proposed four-factor model, providing evidence for the construct distinguishing among SSS, DSS, PsyCap and GSIP.

**Table 3:** Comparison of measurement models.

Model	*χ* ^2^	*df*	Δ*χ*^2^	RMSEA	SRMR	CFI	GFI
SSS, DSS, PsyCap, GSIP	119.25	59	−	0.05	0.03	0.98	0.96
SSS + DSS, PsyCap, GSIP	379.37	62	260.12	0.11	0.05	0.91	0.84
SSS + PsyCap, GSIP, DSS	1079.63	62	960.38	0.20	0.17	0.71	0.62
SSS, DSS + PsyCap, GSIP	1174.02	62	1054.77	0.21	0.19	0.67	0.60
SSS + DSS + PsyCap, GSIP	1373.93	64	1254.68	0.23	0.19	0.63	0.57
SSS + DSS + PsyCap+GSIP	1476.81	65	1357.56	0.23	0.19	0.60	0.53

### Hypothesis testing

#### Relationship between supervisory styles and GSIP

To examine the relationship between supervisory styles and GSIP, a set of regression analyses was conducted using SPSS 26. As shown in Table 4, the regression coefficient between SSS and GSIP was 0.10 (*p* < 0.01), which supported H1a. The effect of DSS on GSIP was also significant (*b* = 0.26, *p* < 0.01); therefore, H1b was also supported.

**Table 4:** The results of the regression analyses of the effects of supervisory styles on GSIP.

Variables	GSIP	
	Model 1	Model 2
Grade	−0.91	−0.90
Discipline	−0.57	−0.49
Gender	1.27^*^	1.21^*^
SSS		0.10^**^
DSS		0.26^**^
*R* ^2^	0.03	0.15
*F*	3.91^**^	13.37^***^

* *p* < 0.05;

** *p* < 0.01;

*** *p* < 0.001 (two tailed).

#### Test for mediation

**Figure 2:**
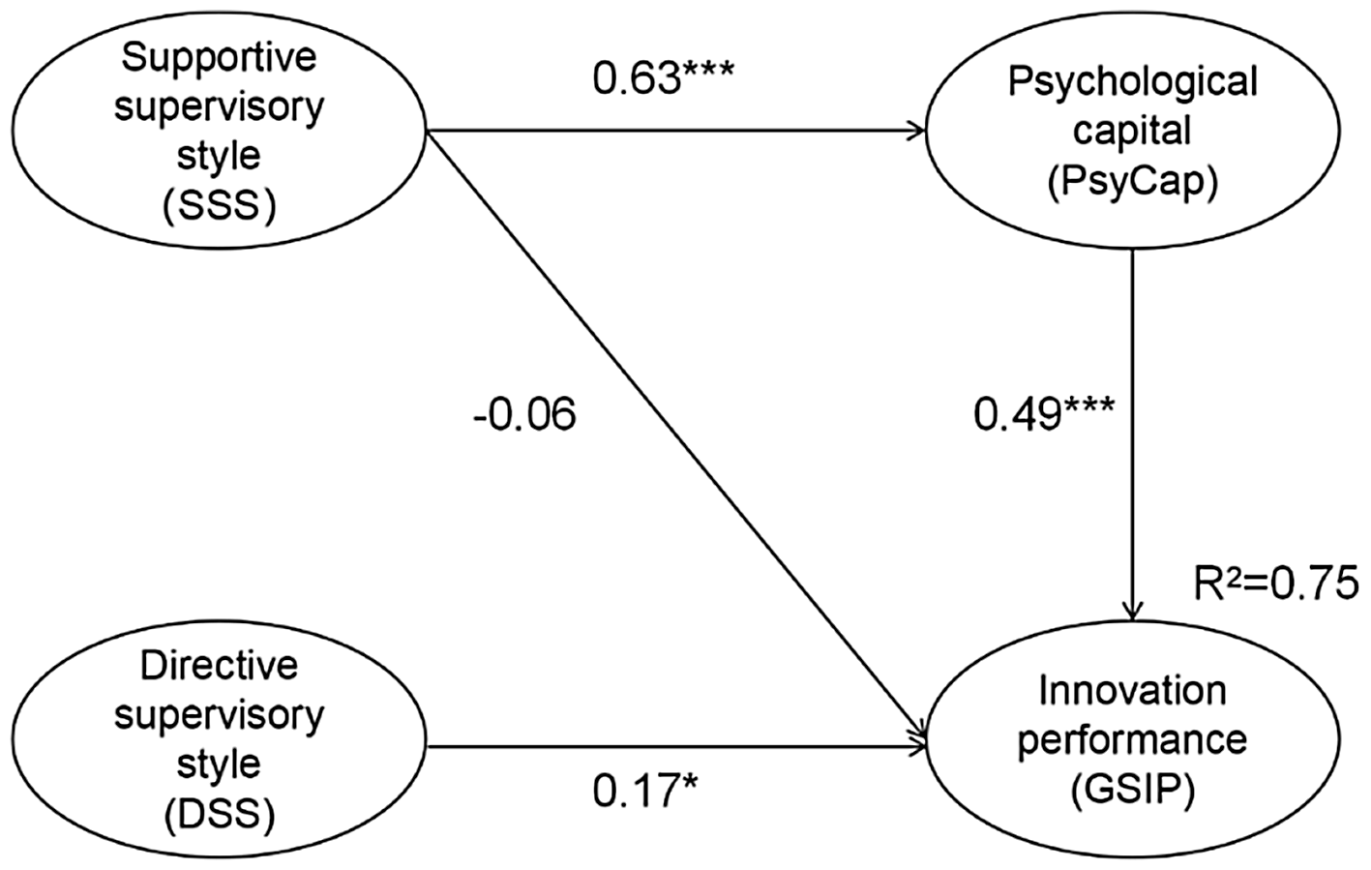
Relationships among the SSS, DSS, PsyCap and GSIP. ^*^p < 0.05, ^***^p < 0.001. Control variables: Gender was coded as male = 1, female = 2. Grade was coded as master’s = 1, PhD student = 2; discipline was coded as humanities and social sciences = 1, science and technology = 2. Fit indices: *χ*^2^/df = 1.90, GFI = 0.95, RMSEA = 0.05, CFI = 0.98, SRMR = 0.05.

According to Figure 2, SSS was significantly and positively associated with PsyCap (*b* = 0.63, *p* < 0.001). Thus, *H2* was accepted. Moreover, PsyCap was significantly and positively correlated with GSIP (*b* = 0.49, p < 0.001), supporting H3.

To explore the mediating effect of PsyCap on the link between SSS and GSIP, a bootstrap analysis with 95% bias-corrected confidence intervals (CIs) and 5,000 resamples was conducted. Table 5 presents the bootstrap result obtained from AMOS 21. Since the *CI* ([0.21, 0.43]) did not include zero, the findings show that PsyCap significantly mediated the effect of SSS on GSIP; thus, H4 was accepted. In addition, the total effect of SSS on GSIP was statistically significant (*CI* [0.04, 0.45]), and the direct effect was nonsignificant (*CI* [−0.25, 0.12]), suggesting that PsyCap fully mediated the effect of SSS on GSIP.

**Table 5:** Indirect effect of SSS on GSIP.

Regression paths	Indirect effects	Boot *SE*	Bias-corrected 95% *CI*	
			Lower	Upper
SSS → PsyCap → GSIP	0.31^***^	0.06	0.21	0.43

*** *p* < 0.001 (two tailed).

#### Test for moderation

To analyse the effects of supervisory styles and HAP on GSIP, two simple moderation analyses classified by supervisory styles were conducted using PROCESS Model 1 (Hayes, 2017). GSIP was entered as the dependent variable; SSS and DSS were entered as independent variables; and HAP was entered as the moderator, with gender, grade and discipline as covariates. All study variables were mean-centered before data analysis. As presented in Table 6, the interaction between DSS and HAP (model 2) was significantly related to GSIP (*b* = 0.20, *p* < 0.001). The interaction variable (SSS × HAP, model 1) had a nonsignificant effect (*b* = 0.01, *p* > 0.05), showing that the impact of SSS on GSIP was not conditional on the level of HAP.

**Table 6:** Moderating role of HAP on the associations between supervisory styles and GSIP in the two models.

Variables	*B*	*SE*	*T*	*P*	95%	
					Lower	Upper
**Model 1**						
SSS	0.08	0.03	3.21	0.00	0.03	0.13
HAP	0.94	0.09	10.91	0.00	0.77	1.11
SSS × HAP	−0.02	0.01	−1.89	0.06	−0.03	0.00
**Model 1**						
DSS	0.20	0.06	3.32	0.00	0.08	0.32
HAP	0.94	0.08	11.14	0.00	0.77	1.10
DSS × HAP	−0.06	0.02	−3.28	0.00	−0.10	−0.02

**Figure 3:**
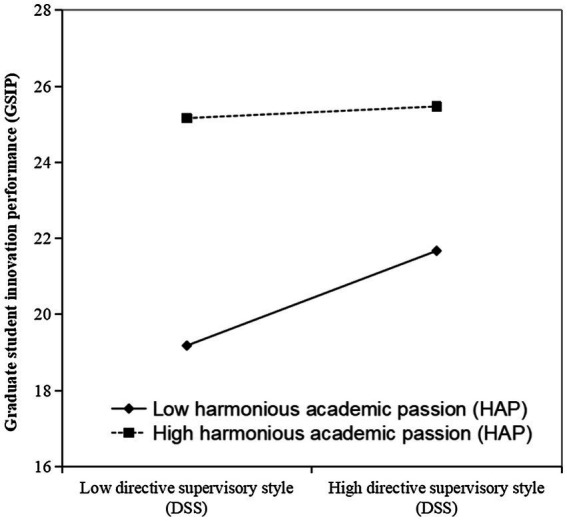
Simple regression lines of DSS on GSIP when students have different levels of HAP.

Figure 3 displays the simple regression lines of DSS on GSIP at low (*M* − *SD*) and high (*M* + *SD*) levels of HAP. The results revealed a stronger positive relationship between DSS and GSIP when students had lower (slope = 0.36, *t* = 4.86, *p* < 0.001) rather than higher (slope = 0.04, *t* = 0.51, *p* > 0.05) levels of HAP.

## Discussion

### Conclusion

Based on the JD-R model and COR theory, this study explored the influence mechanism and boundary conditions of supervisory styles on GSIP using PsyCap as a mediator and HAP as a moderator. Most of the links proposed in this study were supported by the current investigation. Here, the key conclusions are discussed.

First, supervisory style was positively related to GSIP. Specifically, SSS had a considerable impact on GSIP, which is consistent with the conclusions of previous research (Kim and Karau, 2009; Gu et al., 2017; Fan et al., 2019). DSS was positively related to GSIP in the Chinese educational context. By setting deadlines and providing external monitoring, graduate students can decrease laziness, thus boosting their productivity. This finding is in line with the conclusions of previous research, which has also demonstrated that DSS enhances graduate student creativity (Gu et al., 2017). Both results imply that supportive and directive supervisory styles are critical resources that graduate students can employ to engage in innovative behaviors and produce better performance.

Second, SSS significantly enhances graduate students’ PsyCap, which in turn promotes their GSIP. Specific to the initial step of the mediated relationship (between SSS and PsyCap), SSS is positively associated with PsyCap, a finding which is in line with the conclusions of previous research, indicating that supportive climate could provide the fertile soil required for PsyCap to thrive (Ahmed et al., 2017; Um-e-Rubbab et al., 2021). By engaging in various supportive behaviors, such as participative goal setting, the provision of positive feedback, encouragement, empowering students and providing reassurance, supervisors can encourage students to develop greater confidence in their abilities to pursue academic goals, enhance their willingness and ability to design hope pathways, and motivate them to be more optimistic and resilient, thus contributing to their PsyCap (Gupta and Singh, 2014). Furthermore, this study also demonstrated that PsyCap has the potential to predict GSIP, a finding which is consistent with the conclusions of previous studies (Guo et al., 2021). Students with higher PsyCap have more available resources to compensate for the loss of resources in academic research, thus leading to higher performance (Gupta and Singh, 2014; Liu et al., 2020).

Overall, in line with previous research indicating that job resources can increase individuals’ PsyCap, which, as an important psychological resource, can lead to better outcomes (Newman et al., 2014), the findings of this study showed that SSS promotes PsyCap, which in turn contributes to GSIP. This result is also in line with the JD-R model and COR theory, thus suggesting that employees who work in a resourceful environment are likely to develop personal resources that, in turn, facilitate positive outcomes (Schaufeli and Taris, 2014). It should be noted that PsyCap fully mediates the relationship between SSS and GSIP based on the results of this study, which is consistent with previous studies (Luthans et al., 2008; Gu et al., 2017). According to the common saying, “Your teacher can open the door but you must enter by yourself.” If a graduate student lacks the motivation or individual capacity to perform academic research, even the best support would not guarantee a consistent level of success (Luthans et al., 2008). SSS is merely a critical external factor, and its impact on GSIP is affected by students’ internal factors, especially their “will” and “can” (Gu et al., 2017).

Finally, HAP has a significant conditional effect on DSS. Specifically, DSS is more positively related to GSIP when students have lower levels of HAP. This finding is consistent with previous research showing that controlling instruction has a relatively strong impact on the innovative thinking and innovative behavior of graduate students with low individual initiative (Wu et al., 2018). Compared to students with a high level of HAP, students with a low level of HAP have no clear research plan and are more likely to accept tasks assigned by their supervisors, thereby increasing their innovation performance. Contrary to expectations, the moderating role of HAP between SSS and GSIP was not confirmed. This suggests that the positive effect of SSS on GSIP was beyond the specific studied condition.

### Theoretical implications and research contributions

First, the present study enriches the understanding of the influence of supervisory styles on GSIP by introducing PsyCap as the mediator. Supervisory leadership influences graduate students’ innovation *via* complex closer-proximity mediating mechanisms (Fischer et al., 2016; Hughes et al., 2018). However, previous studies on the influence of supervisory styles on innovation and creativity have been limited to an examination of intrinsic motivation (Hughes et al., 2018). It is not possible for graduate students to develop innovation in a psychological vacuum (Liu et al., 2020); instead, PsyCap may have a significant impact on innovation and creativity (Rego et al., 2012; Yan et al., 2020). Based on the JD-R model and COR theory, this study finds that graduate students’ PsyCap, as an important personal resource, fully mediates the relationship between SSS and GSIP, thus highlighting the psychological mechanism in the supervisor leadership process.

Second, this study incorporates HAP as a moderator into the research model, explaining how supervisory styles influence GSIP in a comprehensive way and addressing the request for additional study regarding the significance of individual traits in GSIP (Zacher and Johnson, 2015; Meng et al., 2017).

Finally, the present study employed the JD-R model and COR theory as theoretical foundations to examine the influence of supervisory styles on GSIP in the Chinese academic context. This approach contributes to the SSS literature, as previous researchers have employed the social cognitive theory and Amabile’s componential theory of creativity to explain the relationship between SSS and graduate students’ creativity. The present research expands the application of the JD-R model and COR theory to the field of graduate education. Although they were initially developed to study employees in the workplace, it is reasonable that the two theories can also be utilized as theoretical foundations to predict connections in academic contexts because the relationships between supervisors and graduate students are comparable to workplace relationships (Ahmed et al., 2017; Xia et al., 2021). By employing the JD-R model and COR theory, SSS was found to act as a critical job resource that can enhance graduate students’ PsyCap, which, as a core personal resource, boosts GSIP.

### Practical implications for supervisors

First, to ensure effective supervisor leadership, supervisors should adapt supportive and directive supervisory styles to enrich graduate students’ job resources and personal resources for academic innovation in light of the positive effect of supervisory styles on GSIP. More specifically, supervisors should use the following strategies to help students enhance their PsyCap and innovation skills (Overall et al., 2011): (a) provide task-related assistance, be accessible, and reply to students promptly; (b) encourage students and show empathy toward them as they face research-based challenges, personal difficulties, or confidence crises; and (c) consider students’ viewpoints and allow them to make their own decisions. Furthermore, the findings suggest that students with low HAP might benefit from DSS. Supervisors could use directive strategies, such as providing students with detailed goals and directions and using specific guidance, to help students who have low HAP improve their innovation performance.

Second, PsyCap is state-like in nature and is open to development through training and intentional practice (Luthans et al., 2006; Dello Russo and Stoykova, 2015). Considering the positive effect of PsyCap on GSIP, educational interventions are encouraged that promote graduate students’ PsyCap. On the one hand, nurturing or supporting the social environment, such as through SSS, is likely to generate PsyCap. Thus, supervisors should recognize the significance of their support strategies in strengthening graduate students’ PsyCap. On the other hand, some empirical studies have verified the effectiveness of PsyCap interventions such as daily online self-learning (Da et al., 2020) and academic courses (Gomes da Costa et al., 2021) with respect to enhancing the PsyCap of employees or students. Professional psychological counselling, academic advising programs, or other interventions can help to promote graduate students’ PsyCap.

### Limitations and recommendations

Despite its contributions, the study has several limitations. First, this was a cross-sectional study, which by definition cannot model temporal order; thus, no causal links can be concluded. However, future research can confirm the causal relationships found in this study using longitudinal or time-lagged designs. Second, future studies could use a longitudinal design to examine the reverse causal effects. According to COR theory, initial resource gains lead to future resource gains in a process that is known as gain spirals (Halbesleben et al., 2014), thus implying that reciprocal relationships exist in this context. According to the literature, job characteristics and well-being appear to interact (Simbula et al., 2011). Hence, it is rational to assume that PsyCap and SSS have a reciprocal link with GSIP. Third, because the data used in the study were self-reported, CMB could have skewed the results. Future research could use multistage, multisource designs so that the CMB problem is solved from the onset. Finally, all respondents came from one university in China, limiting the observed variability and external validity. Conducting future research in a range of organizational situations could help to broaden the applicability of the findings.

## Data availability statement

The raw data supporting the conclusions of this article will be made available by the authors, without undue reservation.

## Ethics statement

The studies involving human participants were reviewed and approved by Lanzhou University ethics committee. The patients/participants provided their written informed consent to participate in this study.

## Author contributions

BY: designed the research, conducted data analyses and wrote up the manuscript. SB: was PI on the grant that funded data collection, she also revised, and edited the manuscript. JX: reviewed the literature and revised the manuscript. All authors contributed to the article and approved the submitted version.

## Funding

This work was supported by the National Social Science Fund of China under Grant number BIA200213.

## Conflict of interest

The authors declare that the research was conducted in the absence of any commercial or financial relationships that could be construed as a potential conflict of interest.

## Publisher’s note

All claims expressed in this article are solely those of the authors and do not necessarily represent those of their affiliated organizations, or those of the publisher, the editors and the reviewers. Any product that may be evaluated in this article, or claim that may be made by its manufacturer, is not guaranteed or endorsed by the publisher.
